# Preschool children of parents who screen positive for mental health problems have an increased risk of subsequent mental disorders: Findings from a longitudinal follow-up study in Sweden

**DOI:** 10.1371/journal.pone.0318971

**Published:** 2025-03-12

**Authors:** Sayeda Nazmun Nahar, Anna Fäldt, Anton Dahlberg, Natalie Durbeej

**Affiliations:** Child Health and Parenting (CHAP), Department of Public Health and Caring Sciences, Uppsala University, Uppsala, Sweden; Qatar University College of Nursing, QATAR

## Abstract

Research has shown associations between parental and child mental health problems. However, there is a paucity of Swedish studies on this topic. Investigating this link in a Swedish context could inform preventive interventions aimed at reducing mental health problems in affected families. This longitudinal cohort study aimed to explore the association between parental mental health problems and children’s subsequent mental disorders in Sweden. We used data on children, 3–5 years old, whose mothers (n = 6379) and fathers (n = 6218) had responded to the 12-item General Health Questionnaire for assessment of parental mental health problems using a cut-off of ≥12 points. The children were followed for approximately seven years with regard to subsequent mental disorders, collected from the Swedish National Patient Register. The associations between parental mental health problems and children’s mental disorders were explored through Cox-regression models. In unadjusted Cox regression models, mothers (HR: 1.63, 95% CI: 1.37–1.94) and fathers (HR: 1.36, 95% CI: 1.12–1.65) with mental health problems were more likely to have children diagnosed with a subsequent mental disorder than mothers and fathers with no mental health problems. In adjusted models controlling for children’s emotional and behavioral problems and parental sociodemographic factors, the associations remained significant for mothers’ mental health problems (AHR: 1.33, 95% CI: 1.12–1.59), but not for fathers’ (AHR: 1.14, 95% CI: 0.93–1.40). Children with emotional and behavioral problems, whose parents were single or living apart and whose parents had lower educational levels also had an elevated risk of being diagnosed with a mental disorder. Maternal mental health problems and child emotional and behavioral problems during the preschool years may serve as risk factors for subsequent child mental disorders. Assessment of these problems at child health services in Sweden could facilitate delivery of interventions to promote parental and child mental health.

## Introduction

Mental health problems in children and parents constitute a serious public health issue. Research has proposed that approximately 18% of parents and up to 20% of children and adolescents suffer from mental health problems, i.e., problems ranging from temporary conditions such as sadness, anxiety, and sleep problems to more severe conditions that meet diagnostic criteria for a mental disorder [1,2]. Emotional and behavioral problems, including fear, sadness, anxieties or worries, aggression, defiance, hyperactivity, inattention and lack of impulse control, are some of the most common mental health problems in young children, affecting approximately one in eight children [3]. Further, almost 13% of children are living with a mental disorder [4], i.e., mental health problems that match specific diagnostic criteria. Altogether, early identification of children in need of support for mental health problems is important to promote healthy child development.

Parental mental health problems may act as a stressor for children as parents are central to their lives. More specifically, such problems may lead to negative parenting behaviors, increased family dysfunction, lack of attention to children’s needs, and child mental health problems [5–7]. Research has shown that parental stress is associated with higher levels of child emotional problems [8]. Additionally, maternal depression and anxiety have been linked to children’s depressive and behavioral symptoms as well as attachment issues between the mother and child [9–11]. Moreover, studies have indicated that fathers’ depression and anxiety is associated with an increased risk of emotional and behavioral problems in their children [12,13]. Overall, improved parental mental health is likely to improve children’s wellbeing and life course.

Child mental health problems may negatively impact child educational attainment, social relationships and mental health problems later in life [14,15]. In addition, child mental health problems can influence the development of parental mental health problems [16–19]. Further, socioeconomic deprivation factors, including lower parental education and single marital status have been associated with mental health problems in both parents and their children [20–23]. Research has also shown that ethnic minority parents and children report more mental health problems compared to ethnic parents and children due to unstable living conditions and perceived discrimination [24,25].

In Sweden, mental health problems in children have increased by more than 57% in recent years [26] and the country has higher prevalence rates of mental health problems in children than other European countries [27]. Despite the increasing prevalence of mental health problems among children in Sweden, there is a paucity of research exploring the association between parents’ mental health problems and their offspring’s subsequent mental health problems in a Swedish context. The Swedish child health services provide universal health care to all children from birth to six years of age. Added knowledge on the link between parental mental health problems and young children’s mental health problems could inform preventive interventions aimed at mitigating the burden of mental health problems in Swedish families. The current study aimed to explore the association between parental mental health problems and their children’s subsequent mental disorders in a Swedish context. Based on previous research [8–13], we hypothesized that parents’ mental health problems would be associated with an increased risk of mental disorders in their children.

## Methods

### Study design

This study used a longitudinal cohort study design using data from the “Children and Parents in Focus study” (in short, the “Focus study”). The purpose of the Focus study was to explore the health of preschool-aged children and their parents. It comprised a cohort of children aged 3–5 years for which data were collected between 2013 and 2017 in Uppsala Region, Sweden [28]. In this study, the cohort was followed with regard to registry data from 2013 to 2022, collected from the Swedish National Patient Register (SNPR).

### Participants and procedure

Parents of all children aged 3 to 5 years were recruited from child health centers in Uppsala Region in connection to their child’s visit to the health center for the annual health check-up visit.

An invitation letter and two sets of questionnaires were sent to each child’s home three weeks before the annual visit. Both parents were instructed to complete one questionnaire each. The questionnaires were available in Swedish, English, Arabic, and Somali and included the 12-item General Health Questionnaire (GHQ-12) for assessment of parental mental health problems [29], The Strengths and Difficulties Questionnaire (SDQ) for measurement of children’s emotional and behavioral problems [30] as well as items on child demographics, and parents’ socio-demographic background. The Focus study was conducted during four predefined years, 2013 to 2017 with recruitment starting on August 1, 2013 and ending on October 31, 2017, and obtained data from children at 3, 4, and 5 years of age. Thus, the GHQ-12, the SDQ and items on child demographics, and parents’ socio-demographic background were applied at multiple assessment points. In cases where children were represented more than once, only the earliest data point was retained for this study to explore parental mental health problems when the children were at their youngest age.

Inclusion criteria for the Focus study were that all parents of 3, 4 and 5-year-old children who attended health centers for the annual health check-up visits in Uppsala Region could participate [28]. However, parents who did not understand Swedish or any of the above-mentioned languages were excluded. Over the four study years, more than 28,000 sets of questionnaires were distributed to parents in Uppsala Region and a total of 9496 unique children participated in the Focus study. All children were rated separately by their mothers and fathers. The participation rate in the Focus study varied between 45 and 51% during the four study years [31].

**Fig 1:**
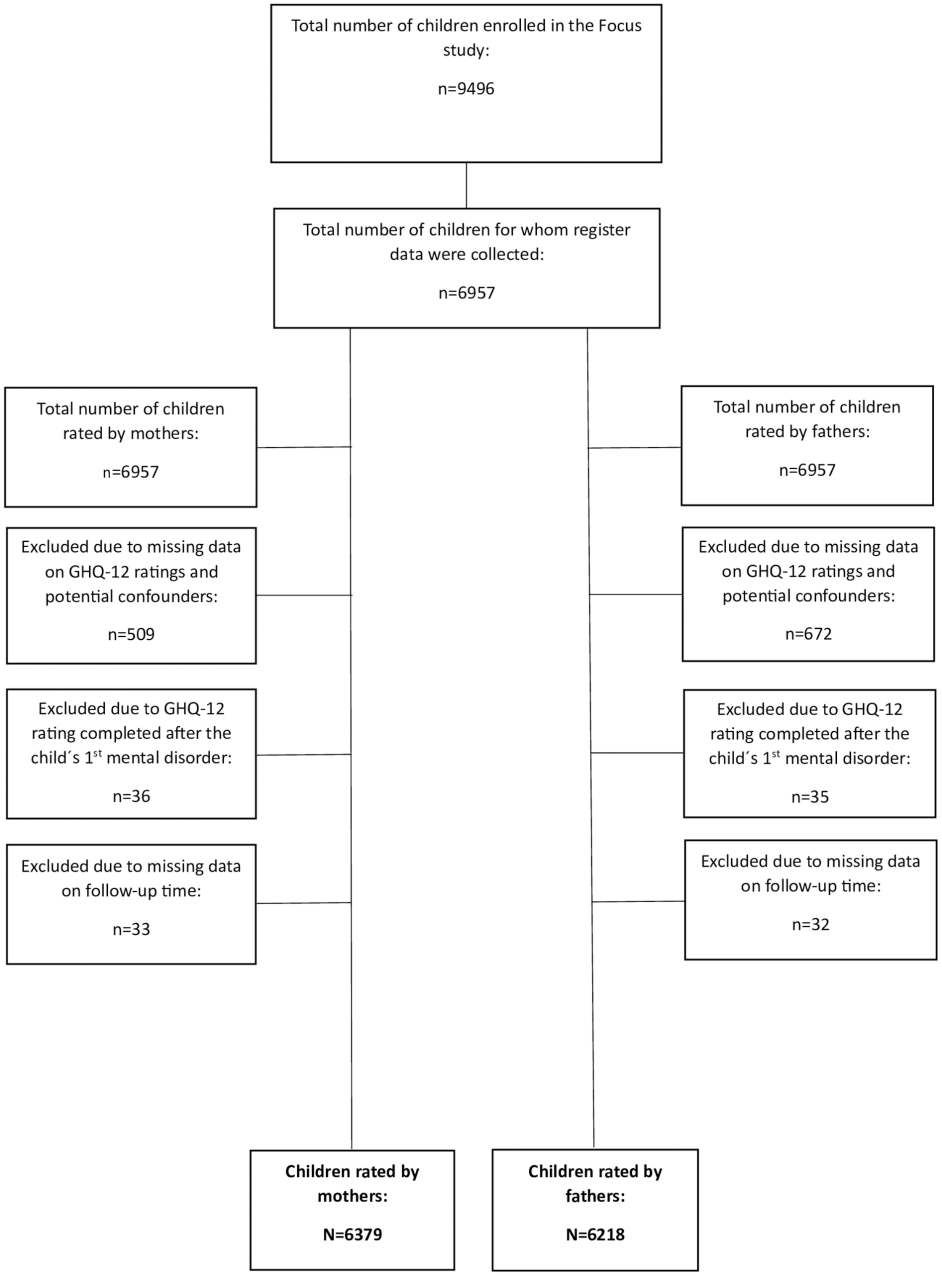
Participant flowchart.

Among the children enrolled in the Focus study, 6957 children from whom data on mental disorders were collected from the SNPR were included in the current study. Among these, 509 ratings from mothers and 672 ratings from fathers were excluded due to missing data on the GHQ-12 and potential confounders selected for this study (see below). In addition, 35 and 36 responses were excluded as the GHQ-12 was completed after the child was diagnosed with a mental disorder to avoid any negative follow-up bias that could interfere with the study’s results. Lastly, 33 and 32 responses from mothers and fathers, respectively, were excluded due to missing data on follow-up time, resulting in a total of 6379 and 6218 unique children who were rated by mothers and fathers, respectively, and who consequently were selected as the study samples of the current study (Fig 1.)

### Data sources

#### Parental mental health problems.

Parental mental health problems were assessed through the 12-item General Health Questionnaire (GHQ-12) [29]. The GHQ-12 is one of the most widely used instruments to assess mental health problems in adult populations. The instrument is designed as a structured and brief self-report questionnaire with items concerning behaviors and functions, e.g., worry, sleeping problems, social dysfunction and loss of confidence, in the last few weeks with four response options ranging from, e.g., “better than usual” to “much worse than usual”. In this study, all items were scored on a Likert scale (0–1–2–3), resulting in a total score ranging from 0 to 36, with higher scores representing higher levels of mental health problems. Further, we used a cut-off of ≥12 points for defining parental mental health problems as it has shown adequate sensitivity (80.4%) and specificity (69.6%) for identifying mental health problems in the Swedish adult general population [32]. The GHQ-12 has also established psychometric properties in adult populations in international research [33].

#### Children’s mental disorders.

Data on children’s mental disorders were obtained from the Swedish National Patient Register (SNPR) through linkage of their social security numbers. The SNPR provides information about in- and specialized outpatient care in Sweden, with the purpose to monitor health trends, improve prevention and treatment of diseases, and monitor and develop health care services [34]. The register was established in 1964, attained complete nationwide coverage in 1987 and has demonstrated high validity and suitability for large-scale population research [35]. For this study, a mental disorder was defined as the child receiving at least one psychiatric diagnosis according to the International Classification of Disease (ICD-10) codes F00–F99 during psychiatric inpatient or specialized outpatient care, as recorded by a physician in the SNPR from 2013 to 2022. Both main and secondary diagnoses were included to define children’s mental disorders. Linkage of the SNPR data with children’s social security numbers and the additional data used in this study was made by the Swedish authority The National Board of Health and Welfare, registry holder of the SNPR.

#### Confounders.

We included confounders on children’s emotional and behavioral problems, and parental socio-demographic factors to adjust for their potential confounding effects. Children’s emotional and behavioral problems were assessed using the parent version of the Strengths and Difficulties Questionnaire (SDQ) [30]. The SDQ is available for children 2–17 years old and covers 25 items that are rated according to a 3-point scale, ranging from 0 to 2 (0 = Not True, 1 = Somewhat True, and 2 = Certainly True). The instrument measures emotional symptoms, conduct problems, peer relationship problems, and hyperactivity/inattention, that comprise a total difficulties score ranging from 0 to 40, with higher scores representing higher levels of emotional and behavioral problems. A separate dimension measuring prosocial behavior is included in the SDQ but was not used in this study since it is not a problem scale intended to capture emotional or behavioral problems. The SDQ is one of the most widely used instruments for assessing emotional and behavioural problems in children and has been subject to research for several decades. The instrument has demonstrated adequate psychometric properties, including construct validity, concurrent validity and internal consistency, when used among school children and preschool children in research across the globe [36–39].

In this study, we used Swedish cut-offs for the SDQ total difficulties score, available for preschool children, to assess emotional and behavioral problems [40]. The following gender- and age specific cut-offs for parents were used: 12 points (3-year-old girls), 11 points (4-year-old girls), 10 points (5-year-old girls), 13 points (3- and 4-year-old boys) and 12 points (5-year-old boys). Data on the confounders pertaining to parental socio-demographic factors were collected through separate self-report items from the Focus study and included: parental country of birth (Sweden or other), parental level of education (higher or lower level), and parental marital status (married/cohabiting or single/living apart/other). The rationale for including the confounders was based on previous research showing associations between such factors, i.e., emotional and behavioral problems, and parental socio-demographic factors including parental education, marital status and country of birth, and mental health problems in both parents and children [16–25].

### Statistical analyses

Means, standard deviations, ranges, frequencies and proportions were used to describe the study samples. Chi-square tests were computed to explore differences in proportions between children with any mental disorder and children with no mental disorder with regard to parental mental health problems and confounders. The relations between parental mental health problems and children’s subsequent mental disorders were explored through unadjusted and adjusted cox regression models of time to the child’s first mental disorder, with days as the underlying time scale. The start date was the date of when the GHQ-12 was completed by both mothers and fathers respectively, and the end date was the date of the first diagnosed mental disorder of their child or 31st of December 2022 if children were not diagnosed with a mental disorder. The mean follow-up time of the children based on mothers’ and fathers’ ratings were 2691 days and 2647 days, respectively, i.e., approximately 7.5 years.

Kaplan-Meier curves were evaluated graphically to explore proportional hazards assumptions. The curves showed that the hazards were proportional for the GHQ-12, the SDQ total difficulties score and the additional confounders. The Cox regression models were performed in two blocks. In the first block, parental mental health problems as measured by the GHQ-12 were entered as the main exposure. In the second block, the SDQ total difficulties score for assessment of children’s emotional and behavioral problems, along with the additional confounders, were added to adjust the results for these variables and to separately explore their associations with the outcome. The outcome any mental disorder was defined as the child receiving at least one psychiatric diagnosis (yes/no) during follow-up.

Given that mothers and fathers provided distinctive information about their mental health problems, socio-demographic position and their children’s emotional and behavioral problems, they were analyzed in separate models. Prior to computing the cox regression models, data were checked for multicollinearity between all independent variables by using variance inflation factor (VIF)-values, a widely used method for this purpose in cases when independent variables are dichotomous in nature [41]. VIF-values ≥10 indicate multicollinearity. No VIF-values exceeded 10, showing that multicollinearity was not present in the data. The results of the regression models are presented as crude and adjusted hazard ratios (HRs and AHRs), along with respective 95% confidence intervals (CIs). Statistical significance was indicated by p-values < 0.05. IBM SPSS version 28 was used as the statistical software for all analyses.

### Ethical considerations

Prior to inclusion in the study, parents or legal guardians of all participating children, aged 3–5 years, gave their written informed consent on behalf of their children. All consent forms were stored in locked cabinets at the Department of Public Health and Caring Sciences, Uppsala university for at least 10 years according to Swedish legislation on data management procedures in research. All parents or legal guardians were informed that participation was voluntary, that confidentiality would be maintained, and that they could withdraw from the study at any time without explanation. The study was approved by the Regional Ethical Review Board in Uppsala/The Ethics Review Authority (document numbers 2012/437 and 2022-06316-02).

## Results

### Participant characteristics

The gender distribution of children was even in both study samples and approximately half of the children were three years old (Table 1). Approximately 9% and 13% of the children had emotional and behavioral problems, as rated by mothers and fathers, respectively. Most mothers and fathers had higher education levels (74.5% and 62.6%), were married or cohabiting (96.2% and 96.5%) and were born in Sweden (85.5% and 85.6%). About 24% of mothers and 18% of fathers had mental health problems. In total, 9.2% of the children were diagnosed with at least one mental disorder during follow-up.

**Table 1:** Description of child demographics, children’s emotional and behavioural problems, parental mental health problems and children’s mental disorders.

	Mothers’ responses (n = 6379)	Fathers’ responses (n = 6218)
**Variables**	**n (%)**	**n (%)**
**Child Gender**		
Boy	3218 (50.4)	3158 (50.8)
Girl	3161 (49.6)	3060 (49.2)
**Child age**		
3 years	3256 (51.1)	3144 (50.6)
4 years	1717 (26.9)	1684 (27.0)
5 years	1406 (22.0)	1390 (22.4)
**Children’s emotional and behavioural problems**		
Identified problems^1^	597 (9.4)	782 (12.6)
Mean SDQ total difficulties score (SD)	6.0 (4.2)	6.6 (4.3)
**Parental education**		
Lower educational level^2^	1628 (25.5)	2324 (37.4)
Higher educational level^3^	4751 (74.5)	3894 (62.6)
**Parental marital status**		
Single/Living apart/other	243 (3.8)	215 (3.5)
Married/Cohabiting	6136 (96.2)	6003 (96.5)
**Parental country of birth**		
Sweden	5455 (85.5)	5365 (86.3)
Outside Sweden	924 (14.5)	853 (13.7)
**Parental mental health problems**		
Identified problems^4^	1543 (24.2)	1125 (18.1)
Mean GHQ-12 total score (SD)	9.4 (4.5)	8.6 (3.9)
**Children’s mental disorders during follow-up**		
Any mental disorder during follow-up^5^	586 (9.2)	574 (9.2)

^1^Defined as scoring above cut-offs for the SDQ total difficulties score

^2^Not completed primary school/completed primary school/completed secondary school

^3^College/university degree

^4^Defined as scoring above or equal to 12 points on the GHQ-12

^5^Defined as any psychiatric diagnoses according to ICD-10 codes F00–F99. Specific diagnose categories included mood disorders (ICD-codes: F30–F39), anxiety disorders (ICD-codes F40–F48), eating disorders (ICD-codes F50), and behavioral and emotional disorders (ICD-codes F90–F98).

### Associations between parental mental problems and children’s mental disorders

Among children diagnosed with any mental disorder, a larger proportion had mothers and fathers with mental health problems (33.1% and 22.6%) than children with no mental disorder (23.3% and 17.6%), respectively (Table 2). Further, children with any diagnosed mental disorder displayed emotional and behavioral problems (24.7% and 25.8%) to a greater extent than children without any disorder (7.8% and 11.2%), according to both parents’ ratings. In addition, among children diagnosed with any mental disorder, larger proportions of children (34.6% and 47.7%) had mothers and fathers with lower education levels than children with no mental disorder (24.6% and 36.3%) as well as fathers born in Sweden (89.2% vs 86.0%). Children diagnosed with any mental disorder also had mothers and fathers who were single or living apart (6.8% and 7.3%) to a greater extent than children with no mental disorder (3.5% and 3.1%).

**Table 2:** Comparisons of children with any mental disorder and no mental disorder during follow-up with regard to child demographics, children’s emotional and behavioural problems, parental socio-demographics, and parental mental health problems. Numbers in bold indicate significant (p < 0.05) associations.

Mothers’ responses	Children with any mental disorder (n = 586)	Children with no mental disorder (n = 5793)	
Variables	n (%)	n (%)	*p* ^1^
**Maternal mental health problems** ^2^			**<0.001**
Identified problems	194 (33.1)	1349 (23.3)	
No identified problems	392 (66.9)	4444 (76.7)	
**Children’s emotional and behavioural problems** ^3^			**<0.001**
Identified problems	145(24.7)	452(7.8)	
No identified problems	441(75.3)	5341(92.2)	
**Maternal education**			**<0.001**
Lower educational level^4^	203(34.6)	1425(24.6)	
Higher educational level^5^	383(65.4)	4368(75.4)	
**Maternal country of birth**			0.050
Sweden	517 (88.2)	4938 (85.2)	
Other	69 (11.8)	855 (14.8)	
**Maternal marital status**			**<0.001**
Single/Living apart/other	40 (6.8)	203 (3.5)	
Married/cohabiting	546 (93.2)	5590 (96.5)	
**Fathers’ responses**	**Children with any mental disorder (n = 574)**	**Children with no mental disorder (n = 5644)**	
**Variables**	**n (%)**	**n (%)**	***p*** ^1^
**Paternal mental health problems** ^2^			**0.003**
Identified problems	130 (22.6)	995 (17.6)	
No identified problems	444 (77.4)	4649 (82.4)	
**Children’s emotional and behavioural problems** ^3^			**<0.001**
Identified problems	148 (25.8)	634 (11.2)	
No identified problems	426 (74.2)	5010 (88.8)	
**Paternal education**			**<0.001**
Lower educational level^4^	274 (47.7)	2050 (36.3)	
Higher educational level^5^	300 (52.3)	3594 (63.7)	
**Paternal country of birth**			**0.033**
Sweden	512 (89.2)	4853 (86.0)	
Other	62(10.8)	791 (14.0)	
**Paternal marital status**			**<0.001**
Single/Living apart/other	42 (7.3)	173 (3.1)	
Married/cohabiting	532 (92.7)	5471 (96.9)	

^1^P-values from corresponding chi-square tests.

^2^Defined as scoring above or equal to 12 points on the GHQ-12

^3^Defined as scoring above cut-offs for the SDQ total difficulties score

^4^Not completed primary school/completed primary school/completed secondary school

^5^College/university degree

In unadjusted Cox regression models, parents with mental health problems were more likely to have children subsequently diagnosed with a mental disorder compared to parents with no mental health problems (Table 3). These associations were shown for both mothers’ (HR: 1.63, 95% CI: 1.37–1.94) and fathers’ (HR: 1.36, 95% CI: 1.12–1.65) ratings. In adjusted Cox regression models, the associations remained significant for mothers’ ratings (AHR: 1.33, 95% CI: 1.12–1.59), but not for fathers’ ratings (AHR: 1.14, 95% CI: 0.93–1.40). In terms of the confounders, children’s emotional and behavioral and emotional problems, as rated by both parents (AHR for mothers: 3.36, 95% CI: 2.77–4.07 and AHR for fathers: 2.50, 95% CI: 2.08–3.05) were associated with a subsequent child mental disorder. In addition, parents with lower educational levels and who were single or living apart had an elevated risk of having children diagnosed with a mental disorder.

**Table 3:** Unadjusted and adjusted cox regression models for exploring the association between parental mental health problems and children’s subsequent mental disorders. Numbers in bold indicate significant (p < 0.05) associations.

Outcome: Any mental disorder during follow-up				
	Mothers’ responses (n = 6379)		Fathers’ responses (n = 6218)	
Independent variables Block I	HR (95% Cl)	*p*	HR (95% Cl)	*p*
**Parental mental health problems**				
No identified problems (ref)				
Identified problems^1^	**1.63 (1.37**–**1.94)**	**< 0.001**	**1.36 (1.12**–**1.65)**	**0.002**
**Independent variables Block II**	**AHR (95% Cl)**	***p***	**AHR (95% Cl)**	***p***
**Parental mental health problems**				
No identified problems (ref)				
Identified problems^1^	**1.33 (1.12**–**1.59)**	**0.001**	1.14 (0.93–1.40)	0.182
**Children’s emotional and behavioural problems**				
No identified problems (ref)				
Identified problems^2^	**3.36 (2.77**–**4.07)**	**< 0.001**	**2.50 (2.08**–**3.05)**	**<0.001**
**Parental education**				
Higher educational level (ref)^3^				
Lower educational level^4^	**1.40 (1.18**–**1.67)**	**<0.001**	**1.40 (1.19**–**1.65)**	**<0.001**
**Paternal country of birth**				
Sweden (ref)				
Other	0.78 (0.60–1.00)	0.055	0.78(0.60–1.02)	0.072
**Paternal marital status**				
Married/Cohabiting(ref)				
Single/Living apart/other	**1.80 (1.30**–**2.48)**	**<0.001**	**2.10 (1.53**–**2.89)**	**<0.001**

^1^Defined as scoring above or equal to 12 points on the GHQ-12.

^2^Defined as scoring above cut-offs for the SDQ total difficulties score.

^3^College/university degree.

^4^Not completed primary school/completed primary school/completed secondary school.

HR, hazards ratio; AHR, adjusted hazards ratio; CI, confidence interval; Ref, reference group.

## Discussion

This study aimed to investigate the association between parental mental health problems and children’s subsequent mental disorders in Sweden. The key findings demonstrated that 24% of mothers and 18% of fathers, respectively, had experienced mental health problems during their children’s preschool years. In addition, 9.2% of the children were diagnosed with at least one mental disorder during the follow-up period. In unadjusted Cox regression models, mothers and fathers with mental health problems were more likely to have children subsequently diagnosed with a mental disorder compared to parents with no mental health problems. In adjusted Cox regression models, the associations remained for mothers’ mental health problems but not for fathers’, suggesting that mothers’ mental health problems might be a particularly important risk factor for their children’s subsequent mental disorders.

Our results resemble previous research findings. For instance, a longitudinal study including families from 22 countries found that parents’ mental disorders predicted their offspring’s mental disorders [42]. Another longitudinal study from Germany found that parents’ depression levels were associated with an earlier onset of depressive disorders in their children [9]. Comparable results were also obtained from a Canadian study showing that paternal mental health problems had less impact than maternal mental health problems on their children’s mental disorders [43]. In our study, children who had identified emotional and behavioral problems during the preschool years also had an elevated risk of being diagnosed with a mental disorder. This finding also aligns with previous research [18,19].

Our findings combined with previous research suggest that parental mental health problems negatively impact child development. In the Swedish health care setting, birth mothers are screened for depression 6–8 weeks postpartum [44]. Although challenges in parenting and life in general affect parents’ mental well-being, parents’ mental health problems are not regularly assessed with valid instruments during the child’s preschool years. Neither are mental health problems in fathers or non-birth parents screened in all Swedish regions. The findings of this paper showed that almost every fifth mother and every fourth father experienced mental health problems during their child’s preschool years. These problems need to be identified and addressed to ensure parental well-being and optimal child development. Further, the association between children’s early emotional and behavioral problems and their subsequent mental disorders, highlights the importance of early identification of emotional and behavioral problems as well as timely interventions targeting these problems. In the Swedish child health services, there are no national policies for screening of emotional and behavioral problems in preschool children. Our results suggest that such a screening could be appropriate.

As mentioned above, our results demonstrated a lower prevalence of mental health problems in fathers compared to mothers, and no association between fathers’ mental health problems according to the GHQ-12, and the children’s mental disorders in the adjusted analysis. These results may have several explanations. One potential explanation could be that maternal mental health problems are more important predictors for children’s mental disorders than paternal mental health problems, as suggested in previous research [43]. In addition, men tend to under-report mental health problems due to gender-related stigma [45] and as far as we are aware, the GHQ-12 cut-off used is not gender specific. Given that men tend to under-report mental health problems due to such stigma, future studies could employ gender-sensitive tools for assessing paternal mental health problems in relation to their offspring’s’ mental disorder in a similar context.

Lastly, children of parents with lower educational levels and parents who were single or living apart also had an elevated risk of being diagnosed with a subsequent mental disorder. These findings have also been demonstrated in previous research [21,22], and suggest that socioeconomically disadvantaged children should be given enhanced attention by the Swedish child health services.

### Strengths and limitations

There are both strengths and limitations to this study. One strength is the large population-based sample and the longitudinal design which enhanced the study’s robustness. In addition, we followed the children and their parents for approximately seven years, which is appropriate for observing the expected outcome, i.e., children’s mental disorders. Another strength is the use of the GHQ-12 and the SDQ for assessing parental mental health problems and child emotional and behavioral problems, respectively, as these instruments have demonstrated good psychometric properties in previous studies on adults and young children [32,37]. Further, the questionnaires were available in multiple languages, meaning that the participants could respond in their preferred languages, ensuring good data quality and a diverse study population. Obtaining separate GHQ-12 ratings from mothers and fathers was a strength, given that comprehensive information on mental health problems in both mothers and fathers were collected. Data on children’s mental disorders were collected from the SNPR, reducing self-reported bias. Finally, by examining the association between parental mental health problems and children’s subsequent mental disorders in Sweden, this study fills a research gap.

Our study did not include important confounders such as parenting style, family dynamics or conflicts, parental unemployment, parental income, or parental support, since data on such factors were inadequate or not collected. Neither did we have data on somatic or chronic conditions in parents or children or on traumatic experiences. Given that such factors may be associated with mental health problems in both parents and children [46–49], this could be considered a limitation. Further, although misclassification of the children’s’ mental disorders was minimized through the use of a high-quality register, i.e., the SPNR, some misclassification could still have occurred since we could not identify children with mental disorders who had not yet been diagnosed or treated, or received a psychiatric diagnosis in primary care. This could be considered as a limitation and may have led to an underestimation of the associations between parents’ mental health problems and their children’s mental disorders. The parental GHQ-12 and SDQ ratings can also have led to bias due to over- or under-reporting of the parental mental health problems and children’s emotional and behavioral problems. The current study did not focus on specific parental mental health problems or child mental disorders as exposures and outcomes, respectively. Thus, future research on this topic could explore these associations in a similar population. Finally, a substantial number of ratings from both mothers and fathers had to be excluded due to missing data on the GHQ-12 and potential confounders, and the majority of parents who rated the children were highly educated and born in Sweden. These circumstances could have led to a selection bias, and therefore, generalization of the findings should be made with caution.

## Conclusions

Maternal mental health problems and child emotional and behavioral problems during preschool years are linked to subsequent mental disorders in children. Our findings suggest the need for the Swedish child health services to implement routine screening for both parental mental health problems and child emotional and behavioral problems. This procedure could facilitate delivery of timely interventions, which in turn could promote mental health in parents and their children.

## References

[1] Stambaugh LF, Forman-Hoffman V, Williams J, Pemberton MR, Ringeisen H, Hedden SL, et al. Prevalence of serious mental illness among parents in the United States: results from the National Survey of Drug Use and Health, 2008–2014. Ann Epidemiol. 2017;27(3):222–4. doi: 10.1016/j.annepidem.2016.12.005 28081894

[2] Castelpietra G, Knudsen AKS, Agardh EE, Armocida B, Beghi M, Iburg KM, et al. The burden of mental disorders, substance use disorders and self-harm among young people in Europe, 1990–2019: Findings from the Global Burden of Disease Study 2019. Lancet Reg Health Eur. 2022;16:100341. doi: 10.1016/j.lanepe.2022.100341 35392452 PMC8980870

[3] Egger HL, Angold A. Common emotional and behavioral disorders in preschool children: Presentation, nosology, and epidemiology. J Child Psychol Psychiatry. 2006;47(3–4):313–37. doi: 10.1111/j.1469-7610.2006.01618.x 16492262

[4] Keeley B. The State of the World’s Children 2021: on my mind: promoting, protecting and caring for children’s mental health. New York: UNICEF; 2021.

[5] Elgar FJ, Mills RS, McGrath PJ, Waschbusch DA, Brownridge DA. Maternal and paternal depressive symptoms and child maladjustment: The mediating role of parental behavior. J Abnorm Child Psychol. 2007;35(6):943–55. doi: 10.1007/s10802-007-9145-0 17577659

[6] Wilson S, Durbin CE. Effects of paternal depression on fathers’ parenting behaviors: a meta-analytic review. Clin Psychol Rev. 2010;30(2):167–80. doi: 10.1016/j.cpr.2009.10.007 19926376

[7] Wilson S, Durbin CE. Parental personality disorder symptoms are associated with dysfunctional parent-child interactions during early childhood: a multilevel modeling analysis. Personal Disord. 2012;3(1):55–65. doi: 10.1037/a0024245 22448861

[8] Löchner J, Ulrich SM, Lux U. The impact of parents’ stress on parents’ and young children’s mental health—short‐ and long‐term effects of risk and resilience factors in families with children aged 0–3 in a representative sample. Stress Health. 2024;40(4):e3400. 38625815 10.1002/smi.3400

[9] Lieb R, Isensee B, Höfler M, Pfister H, Wittchen H-U. Parental major depression and the risk of depression and other mental disorders in offspring: a prospective-longitudinal community study. Arch Gen Psychiatry. 2002;59(4):365–74. doi: 10.1001/archpsyc.59.4.365 11926937

[10] Choi KW, Houts R, Arseneault L, Pariante C, Sikkema KJ, Moffitt TE. Maternal depression in the intergenerational transmission of childhood maltreatment and its sequelae: testing postpartum effects in a longitudinal birth cohort. Dev Psychopathol. 2019;31(1):143–56. doi: 10.1017/S0954579418000032 29562945 PMC6033315

[11] Glasheen C, Richardson GA, Fabio A. A systematic review of the effects of postnatal maternal anxiety on children. Arch Womens Ment Health. 2010;13(1):61–74. doi: 10.1007/s00737-009-0109-y 19789953 PMC3100191

[12] Ramchandani P, Psychogiou L. Paternal psychiatric disorders and children’s psychosocial development. Lancet. 2009;374(9690):646–53. doi: 10.1016/S0140-6736(09)60238-5 19411102

[13] Ramchandani P, Stein A, Evans J, O’Connor TG; ALSPAC Study Team. Paternal depression in the postnatal period and child development: a prospective population study. Lancet. 2005;365(9478):2201–5. doi: 10.1016/S0140-6736(05)66778-5 15978928

[14] Kieling C, Baker-Henningham H, Belfer M, Conti G, Ertem I, Omigbodun O, et al. Child and adolescent mental health worldwide: evidence for action. Lancet. 2011;378(9801):1515–25. doi: 10.1016/S0140-6736(11)60827-1 22008427

[15] Baranne ML, Falissard B. Global burden of mental disorders among children aged 5–14 years. Child Adolesc Psychiatry Ment Health. 2018;12(1):1–9. 29682005 10.1186/s13034-018-0225-4 PMC5896103

[16] Campbell SB, Shaw DS, Gilliom M. Early externalizing behavior problems: toddlers and preschoolers at risk for later maladjustment. Dev Psychopathol. 2000;12(3):467–88. doi: 10.1017/s0954579400003114 11014748

[17] Salari R, Wells MB, Sarkadi A. Child behaviour problems, parenting behaviours and parental adjustment in mothers and fathers in Sweden. Scand J Public Health. 2014;42(7):547–53. doi: 10.1177/1403494814541595 25005931

[18] Finsaas MC, Bufferd SJ, Dougherty LR, Carlson GA, Klein DN. Preschool psychiatric disorders: homotypic and heterotypic continuity through middle childhood and early adolescence. Psychol Med. 2018;48(13):2159–68. doi: 10.1017/S0033291717003646 29335030 PMC6047937

[19] Dougherty LR, Smith VC, Bufferd SJ, Kessel E, Carlson GA, Klein DN. Preschool irritability predicts child psychopathology, functional impairment, and service use at age nine. J Child Psychol Psychiatry. 2015;56(9):999–1007. doi: 10.1111/jcpp.12403 26259142 PMC4531384

[20] Fitzsimons E, Goodman A, Kelly E, Smith JP. Poverty dynamics and parental mental health: determinants of childhood mental health in the UK. Soc Sci Med. 2017;175:43–51. doi: 10.1016/j.socscimed.2016.12.040 28056382 PMC5293621

[21] Meyrose A-K, Klasen F, Otto C, Gniewosz G, Lampert T, Ravens-Sieberer U. Benefits of maternal education for mental health trajectories across childhood and adolescence. Soc Sci Med. 2018;202:170–8. doi: 10.1016/j.socscimed.2018.02.026 29554584

[22] Choi W-J, Kwon H-J, Lim MH, Lim J-A, Ha M. Blood lead, parental marital status and the risk of attention-deficit/hyperactivity disorder in elementary school children: a longitudinal study. Psychiatry Res. 2016;236:42–6. doi: 10.1016/j.psychres.2016.01.002 26774190

[23] Caksen H. The effects of parental divorce on children. Psychiatriki. 2022;33(1):81–2. doi: 10.22365/jpsych.2021.040 34860682

[24] Whitaker L, Cameron C, Hauari H, Hollingworth K, O’Brien M. What family circumstances, during COVID-19, impact on parental mental health in an inner city community in London? Front Psychiatry. 2021;12:725823. doi: 10.3389/fpsyt.2021.725823 34975559 PMC8716836

[25] Adriaanse M, Veling W, Doreleijers T, van Domburgh L. The link between ethnicity, social disadvantage and mental health problems in a school-based multiethnic sample of children in the Netherlands. Eur Child Adolesc Psychiatry. 2014;23(11):1103–13. doi: 10.1007/s00787-014-0564-5 24927803

[26] Cosma A, Abdrakhmanova S, Taut D, Schrijvers K, Catunda C, Schnohr C. A focus on adolescent mental health and well-being in Europe, Central Asia, and Canada: Health Behaviour in School-aged Children international report from the 2021/2022 survey. Copenhagen: WHO Regional Office for Europe; 2023.

[27] Inchley J, Currie DB, Budisavljevic S, Torsheim T, Jastad A, Cosma A, et al. Spotlight on adolescent health and wellbeing: findings from the 2017/2018 Health Behaviour in School-aged Children (HBSC) survey in Europe and Canada. Copenhagen: WHO Regional Office for Europe; 2020.

[28] Salari R, Fabian H, Prinz R, Lucas S, Feldman I, Fairchild A, et al. The Children and Parents in Focus project: a population-based cluster-randomised controlled trial to prevent behavioural and emotional problems in children. BMC Public Health. 2013;13(1):1–8. 24131587 10.1186/1471-2458-13-961 PMC4016486

[29] Goldberg DP, Williams P. A user’s guide to the General Health Questionnaire. Windsor: NFER-Nelson; 1988.

[30] Goodman R. The Strengths and Difficulties Questionnaire: a research note. J Child Psychol Psychiatry. 1997;38(5):581–6. doi: 10.1111/j.1469-7610.1997.tb01545.x 9255702

[31] Fält E, Salari R, Fabian H, Sarkadi A. Facilitating implementation of an evidence-based method to assess the mental health of 3–5-year-old children at Child Health Clinics: a mixed-methods process evaluation. PLoS One. 2020;15(6):e0234383. doi: 10.1371/journal.pone.0234383 32520968 PMC7286525

[32] Lundin A, Hallgren M, Theobald H, Hellgren C, Torgén M. Validity of the 12-item version of the General Health Questionnaire in detecting depression in the general population. Public Health. 2016;136:66–74. doi: 10.1016/j.puhe.2016.03.005 27040911

[33] Wojujutari AK, Idemudia ES, Ugwu LE. The evaluation of the General Health Questionnaire (GHQ-12) reliability generalization: a meta-analysis. PLoS One. 2024;19(7):e0304182. doi: 10.1371/journal.pone.0304182 39018280 PMC11253975

[34] Laugesen K, Ludvigsson JF, Schmidt M, Gissler M, Valdimarsdottir UA, Lunde A, et al. Nordic health registry-based research: a review of health care systems and key registries. Clin Epidemiol. 2021;13:533–54. doi: 10.2147/CLEP.S314959 34321928 PMC8302231

[35] Ludvigsson JF, Andersson E, Ekbom A, Feychting M, Kim JL, Reuterwall C, et al. External review and validation of the Swedish national inpatient register. BMC Public Health. 2011;11:450. doi: 10.1186/1471-2458-11-450 21658213 PMC3142234

[36] Stone LL, Otten R, Engels RC, Vermulst AA, Janssens JM. Psychometric properties of the parent and teacher versions of the strengths and difficulties questionnaire for 4-to 12-year-olds: a review. Clin Child Fam Psychol Rev. 2010;13(3):254–74. doi: 10.1007/s10567-010-0071-2 20589428 PMC2919684

[37] Goodman A, Goodman R. Strengths and difficulties questionnaire as a dimensional measure of child mental health. J Am Acad Child Adolesc Psychiatry. 2009;48(4):400–3. doi: 10.1097/CHI.0b013e3181985068 19242383

[38] Gustafsson BM, Gustafsson PA, Proczkowska-Björklund M. The Strengths and Difficulties Questionnaire (SDQ) for preschool children—a Swedish validation. Nord J Psychiatry. 2016;70(8):567–74. doi: 10.1080/08039488.2016.1184309 27241951

[39] Dahlberg A, Ghaderi A, Sarkadi A, Salari R. SDQ in the hands of fathers and preschool teachers—psychometric properties in a non-clinical sample of 3–5-year-olds. Child Psychiatry Hum Dev. 2019;50(1):132–41. doi: 10.1007/s10578-018-0826-4 29959588 PMC6373308

[40] Dahlberg A, Fält E, Ghaderi A, Sarkadi A, Salari R. Swedish norms for the Strengths and Difficulties Questionnaire for children 3–5 years rated by parents and preschool teachers. Scand J Psychol. 2020;61(2):253–61. doi: 10.1111/sjop.12606 31833080 PMC7079007

[41] Craney TA, Surles JG. Model-dependent variance inflation factor cutoff values. Qual Eng. 2002;14(3):391–403. doi: 10.1081/qen-120001878

[42] McLaughlin KA, Gadermann AM, Hwang I, Sampson NA, Al-Hamzawi A, Andrade LH, et al. Parent psychopathology and offspring mental disorders: results from the WHO World Mental Health Surveys. Br J Psychiatry. 2012;200(4):290–9. doi: 10.1192/bjp.bp.111.101253 22403085 PMC3317036

[43] Jones SL, Caccese C, Davis KP, Lew J, Elgbeili G, Herba CM, et al. Longitudinal associations between paternal mental health and child behavior and cognition in middle childhood. Front Psychol. 2023;14:1218384. doi: 10.3389/fpsyg.2023.1218384 38022974 PMC10646505

[44] Fransson E. National guidelines for nursing in child health care. Stockholm: Stockholm Region; 2024.

[45] Sigmon ST, Pells JJ, Boulard NE, Whitcomb-Smith S, Edenfield TM, Hermann BA, et al. Gender differences in self-reports of depression: The response bias hypothesis revisited. Sex Roles. 2005;53(5–6):401–11. doi: 10.1007/s11199-005-6762-3

[46] Wesseldijk LW, Dieleman G, van Steensel F, Bartels M, Hudziak J, Lindauer R, et al. Risk factors for parental psychopathology: a study in families with children or adolescents with psychopathology. Eur Child Adolesc Psychiatry. 2018;27:1575–84. 29644474 10.1007/s00787-018-1156-6 PMC6245117

[47] Lansford JE, Chang L, Dodge KA, Malone PS, Oburu P, Palmérus K, et al. Physical discipline and children’s adjustment: cultural normativeness as a moderator. Child Dev. 2005;76(6):1234–46. doi: 10.1111/j.1467-8624.2005.00847.x 16274437 PMC2766084

[48] Van Loon LMA, Van de Ven MOM, Van Doesum KTM, Witteman CLM, Hosman CMH. The relation between parental mental illness and adolescent mental health: the role of family factors. J Child Fam Stud. 2014;23(7):1201–14. doi: 10.1007/s10826-013-9781-7

[49] Grant A, McCartan C, Davidson G, Bunting L, Cameron J, McBride O, et al. Prevalence and risk factors of parental mental health problems: a cross-sectional study. Int J Ment Health Nurs. 2024;33(6):2090–101. doi: 10.1111/inm.13365 38867456

